# Determining the value contribution of selexipag for the treatment of pulmonary arterial hypertension (PAH) in Spain using reflective multi-criteria decision analysis (MCDA)

**DOI:** 10.1186/s13023-018-0966-4

**Published:** 2018-12-10

**Authors:** Alberto Jiménez, Arantza Ais, Amélie Beaudet, Alicia Gil

**Affiliations:** 1 Hospital Virgen de las Nieves, Granada, Spain; 2 Hospital Gregorio Marañón, Madrid, Spain; 3 Actelion Pharmaceuticals Ltd, Allschwil, Switzerland; 4 Omakase Consulting S.L., Madrid, Spain

**Keywords:** Multi-criteria decision analysis, MCDA, Rare disease, Pulmonary arterial hypertension, Selexipag, Iloprost

## Abstract

**Background:**

Pulmonary Arterial Hypertension (PAH) is a chronic rare disease that can lead to serious cardiovascular problems and death. Additional treatments that increase effectiveness, that are safe and with a convenient administration that improve outcomes and quality of life for patients are needed. The aim of this study was to assess the value contribution of the new, oral prostacyclin receptor agonist, selexipag, for PAH treatment in Spain through reflective Multicriteria Decision Analysis (MCDA) methodology.

**Methods:**

A comprehensive literature review was performed to develop an evidence matrix, composed of twelve quantitative criteria and four contextual criteria, based on an EVIDEM MCDA framework adapted to orphan drugs evaluation by the Spanish region of Catalonia. Quantitative performance scores, qualitative impact of contextual criteria and individual reflections from stakeholders were collected for each MCDA framework criteria. The value contribution of selexipag to PAH treatment compared to inhaled iloprost was calculated.

**Results:**

Oral selexipag for PAH treatment was considered as a treatment which adds value, compared to iloprost, in the following MCDA quantitative criteria: comparative efficacy, patient reported outcomes, preventive benefit, therapeutic benefit, other medical costs and other non-medical costs, without significant differences in safety profile but with a higher acquisition cost than inhaled iloprost.

**Conclusions:**

Selexipag was considered to provide value to PAH treatment. It was perceived as an intervention indicated for a severe rare disease with high unmet needs, supported by high quality clinical evidence. When compared to inhaled iloprost, oral selexipag has demonstrated improvements in efficacy and patient reported outcomes, with a similar safety profile and some additional costs.

Reflective MCDA provided a standardised, transparent approach to evaluate multiple criteria relating to the overall value contribution of selexipag to PAH treatment facilitating decision-making.

## Background

Pulmonary Arterial Hypertension (PAH) is a chronic rare disease which causes a progressive right ventricular dysfunction that can lead to severe right heart cardiac insufficiency and death [1]. PAH prevalence is estimated at 15–50 cases per million of inhabitants with a median survival time after diagnosis of 2.8 years [2, 3] when untreated. Current treatments for PAH aim to improve the physical function and quality of life of patients, but there is no cure to date. Drugs are available for three key pathogenic pathways associated with PAH: the nitric oxide pathway, the endothelin pathway, and the prostacyclin pathway. Phosphodiesterase type 5 inhibitors (PDE5i) and endothelin receptor antagonists (ERAs) are used as first line treatments mainly due to their convenient oral administration and the long clinical experience [4].

The severity of PAH is typically determined according to the classification of the World Health Organization (WHO Functional Classification (FC)) for PAH [5], which classify patients into four different classes: FC I to FC IV, where the higher classes indicates more severe disease status. When untreated, median survival is only 6 months for patients in WHO FC IV, compared with 2.5 years for those in WHO FC III, and 6 years for those in WHO FC I and II [5]. Therapies targeting the prostacyclin pathway are recommended for patients in FC II-IV [1], but their use has been limited by their mode of administration [6]: continuous parenteral administration or frequent inhaled administration (6–9 times daily) [7]. Moreover, they were approved only based on short-term, monotherapy studies, as these were the first treatments available. Therefore, there is a need for an effective, safe and convenient treatment acting on the prostacyclin pathway in order to prevent disease progression and a higher WHO FC classification.

Selexipag is a new selective agonist of prostacyclin receptor (IP) which is administered orally twice a day. Stimulation of IP by selexipag and its active metabolite causes vasodilatory, antiproliferative and antifibrotic effects. Selexipag is indicated for the long-term treatment of PAH in adult patients with FC II-III, as combination therapy in patients insufficiently controlled with an ERA and/or a PDE-5 inhibitor, or as monotherapy in patients who are not candidates for these treatments [8]. The efficacy of selexipag has been demonstrated in a large (*n* = 1156), placebo-controlled, long-term phase III clinical trial (GRIPHON study) [9]. Selexipag significantly reduced the risk of occurrence of morbidity-mortality events by 40%, the risk of hospitalisation by 33% and disease progression by 64%. The most frequent treatment-related adverse events (AEs) reported were headache, diarrhoea, jaw pain and nausea [10]. According to current clinical practice in PAH in Spain [1], selexipag could be positioned as an alternative to iloprost, the only non-parenteral drug acting on the prostacyclin pathway available in Spain which is administered by inhalation, in 20-min sessions, between 6 and 9 times daily [10].

Healthcare reimbursement decisions for drugs indicated to treat rare diseases are challenging. Thus, assessment of the value and the most adequate positioning within healthcare systems of a drug indicated for the treatment of a rare disease should be holistic, requiring a broader perspective, not limited to the traditional criteria of efficacy, safety and cost [11, 12]. Reflective multi-criteria decision analysis (MCDA) offers a framework with which to make complex healthcare decision-making problems into a comprehensive set of criteria relevant for establishing the value of a drug in an explicit, holistic and systematic way [13, 14].

The aim of this study was to assess the value contribution of selexipag relative to inhaled iloprost for PAH treatment through reflective MCDA methodology from the perspective of all relevant key stakeholders, including evaluators, clinicians, regional decision makers, hospital pharmacists and patients, in Spain.

## Methods

### Study design

The study was designed according to MCDA methodology [13, 14], using the criteria from the MCDA framework specifically adapted for the appraisal of drugs indicated to treat rare diseases in Spain [15]. Inhaled iloprost was the chosen comparator [1]. A literature review was conducted to obtain relevant information on the disease and its current management in Spain as well as relevant evidence for both compared products. Information was structured into an evidence matrix. This matrix was scored by a broad multidisciplinary panel of Spanish stakeholders involved in healthcare decision-making. Scores were analysed quantitatively. Comments and reflections behind experts’ scores were collected in a qualitative manner.

### Literature review

A literature review was conducted to identify available evidence for selexipag and inhaled iloprost. Available evidence was used to create the MCDA evidence matrix.

Two types of documents were searched: published evidence in biomedical databases and specific product evaluations for selexipag and inhaled iloprost by official healthcare evaluation bodies.

Published evidence was searched in PubMed and MEDES (Medicina en Español) [16] databases in order to answer the following search question: What is the available evidence on epidemiology, health outcomes, unmet needs and economic consequences for the evaluation of drugs indicated for treatment of PAH in Spain? The PICOTS (population, intervention, comparison, outcomes, time span and studies) search strategy was used [17, 18]. The literature review in biomedical databases included published studies from 2007 to 2017.

Specific product evaluations for selexipag and inhaled iloprost were searched in official European and Spanish healthcare evaluation bodies’ webpages [e.g. European Medicines Agency (EMA) [19], Spanish Medicines Agency (AEMPS) [20] and Spanish regional and hospital evaluations [21]]. All evaluations found were included, regardless of the date of publication.

At the time of the study start, the reimbursed price for selexipag had not yet been established in Spain, so a hypothetical price was used. The approved list price [22] was later used in the second phase of the study.

### Reflective MCDA tool and evidence matrix development

The MCDA framework used was the one adapted and adopted for the appraisal of drugs indicated to treat rare diseases developed by the Catalonian Regional Healthcare Service (Catsalut) [15]. This adapted MCDA framework is based on the EVIDEM framework (version 4.0) [23] and composed of a total of 16 criteria (Table 1). These criteria are structured into two sections: the MCDA Core Model composed of 12 quantitative criteria focused on product evaluation and the MCDA Contextual Tool composed of 4 contextual criteria focused on the consideration of the context surrounding decision-making).

**Table 1:** MCDA EVIDEM framework version 4.0 adapted to evaluation of medicines indicated for rare diseases

QUANTITATIVE CRITERIA: MCDA Core Model	
DOMAIN: Disease needs	
Disease severity	
Unmet needs	
DOMAIN: Comparative outcomes of interventions (selexipag vs iloprost)	
Comparative efficacy/effectiveness	
Comparative safety/tolerability	
Comparative patient-perceived health/patient reported outcomes (PRO)	
DOMAIN: Type of benefit provided by selexipag	
Type of preventive benefit	
Type of therapeutic benefit	
DOMAIN: Comparative economic consequences of interventions (selexipag vs iloprost)	
Comparative cost of intervention	
Comparative other medical costs	
Comparative other non-medical costs	
DOMAIN: Knowledge about selexipag	
Quality of evidence	
Expert consensus/clinical practice guidelines (CPG)	
CONTEXTUAL CRITERIA: MCDA Contextual Tool	
Population priorities and access	
Common goals and specific interests	
System capacity and appropriate use of intervention	
Opportunity costs and affordability	

To compare selexipag with inhaled iloprost in the comparative criteria of the MCDA framework, data from randomised control trials from both interventions were used and compared in a descriptive manner.

### Expert panel design and conduct of the study

The study was conducted with a multidisciplinary panel of 28 people involved in the management of PAH treatments and decision-making in Spain, including evaluators, clinicians, regional decision makers, hospital pharmacists from different regions and a patient representative in order to collect insights from a broad range of perspectives.

The study was conducted in two phases: a first face to face meeting with seven experts in the evaluation and management of PAH treatments in Spain, where participants received prior training on reflective MCDA methodology. The experts scored the evidence matrix and the reflections behind the score results were collectively discussed to assess the value contribution of selexipag. This first phase was conducted in November 2016.

The second phase of the study, performed in June 2017, had the objective of validating the results obtained in the first phase and improve the robustness of the results by increasing the sample size. This second part involved 21 participants and was performed using an online platform (Jotform) [24]. Prior to study start, participants received on-line training on reflective MCDA methodology.

### Data analysis

Data were collected from each participant, transferred to a common database and analysed with Microsoft Excel. A descriptive analysis of each criterion was conducted. The evaluation of quantitative criteria was performed using a direct rating scale, which varied depending on the type of criteria: from 0 to + 5 for non-comparative criteria and from − 5 to + 5 for comparative criteria. Results of quantitative criteria scoring were given in the form of mean ± standard deviation (SD). The value contribution (VCx) of each quantitative criterion was calculated as the product of its normalised weight (Wx, ∑ Wx = 1) and standardised score (Sx = score/5). Total value estimate (VT) is the sum of all criteria value contributions:

$$ \mathrm{VT}=\sum \limits_{x=1}^{\mathrm{n}}{\mathrm{VC}}_x=\sum \limits_{x=1}^n\left({\mathrm{W}}_x\times {\mathrm{S}}_x\right) $$

The evaluation of contextual criteria was performed on a qualitative scale with three options (positive, neutral or negative impact) and scores were transformed into a numerical scale (+ 1, 0 and − 1 points respectively).

A two-way ANOVA test of the score means for each criterion obtained from the two study phases was carried out to assess potential differences between scores from both phases of the study. All the quantitative criteria, except for “cost of intervention” (because the price used in both phases of the study was different) were considered in this analysis.

### Treatment of inconsistencies

Scores attributed to human error or failure to understand the criteria (e.g.: a higher score assigned to the criteria “comparative cost of the intervention” for an intervention that is more expensive than the comparator) were considered as inconsistencies, excluded from the analysis and replaced using the mean substitution.

## Results

### Literature review

**Fig. 1:**
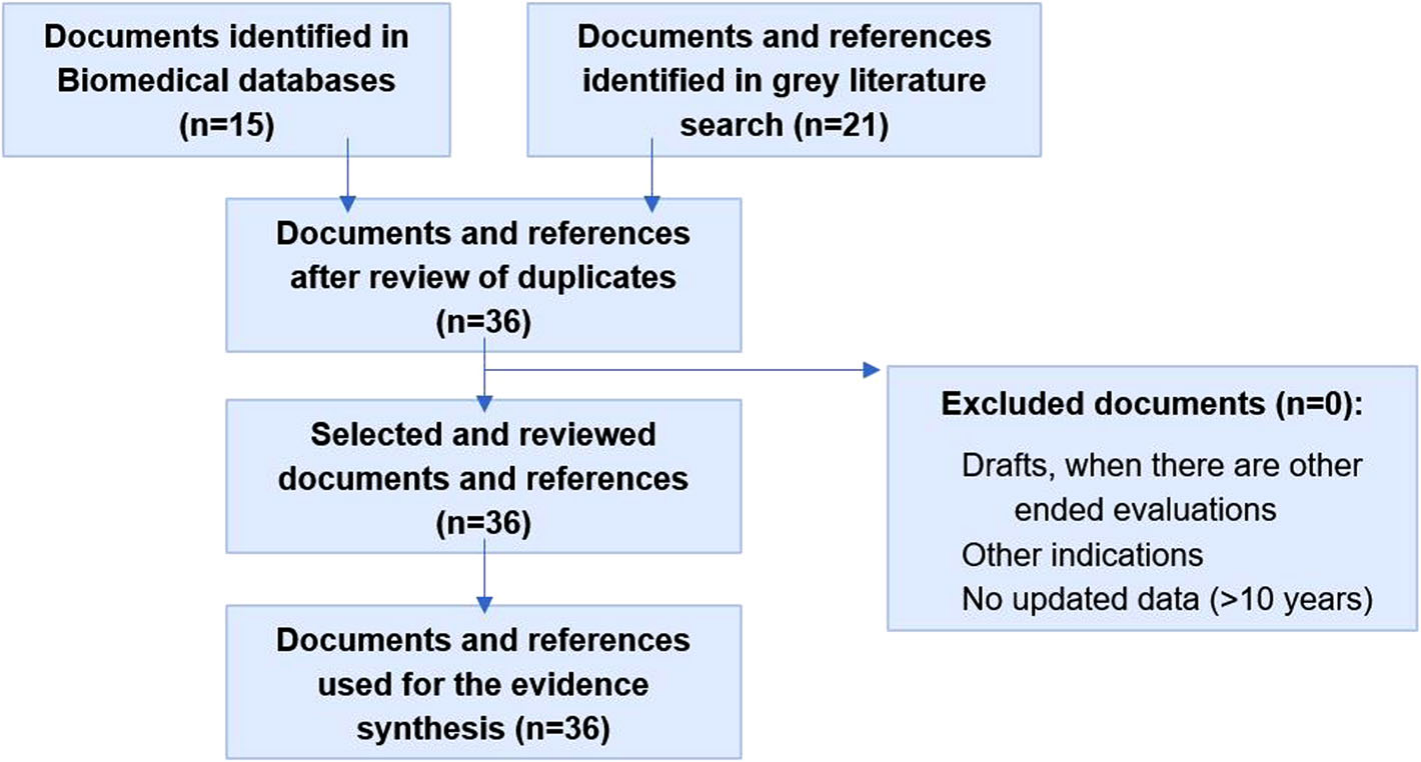
PRISMA diagram [34] of the literature review performed

The evidence matrix was constructed with a total of 36 identified references (Fig. 1) found in biomedical databases (*n* = 15), documents from official sources (i.e.: EMA, AEMPS) (*n* = 2), regional and hospital evaluations (*n* = 1), clinical guidelines or protocols (*n* = 2) and online grey literature (*n* = 16).

The literature review for the first phase was conducted in October 2016 and then updated in May 2017 for the second phase. To calculate the budget impact of selexipag, and in the absence of any public data source, the number of target patients used was based on estimates provided by Actelion Pharmaceuticals Ltd. (personal communication; reference on file).

### Performance scores based on evidence and participants’ insights on selexipag

**Fig. 2:**
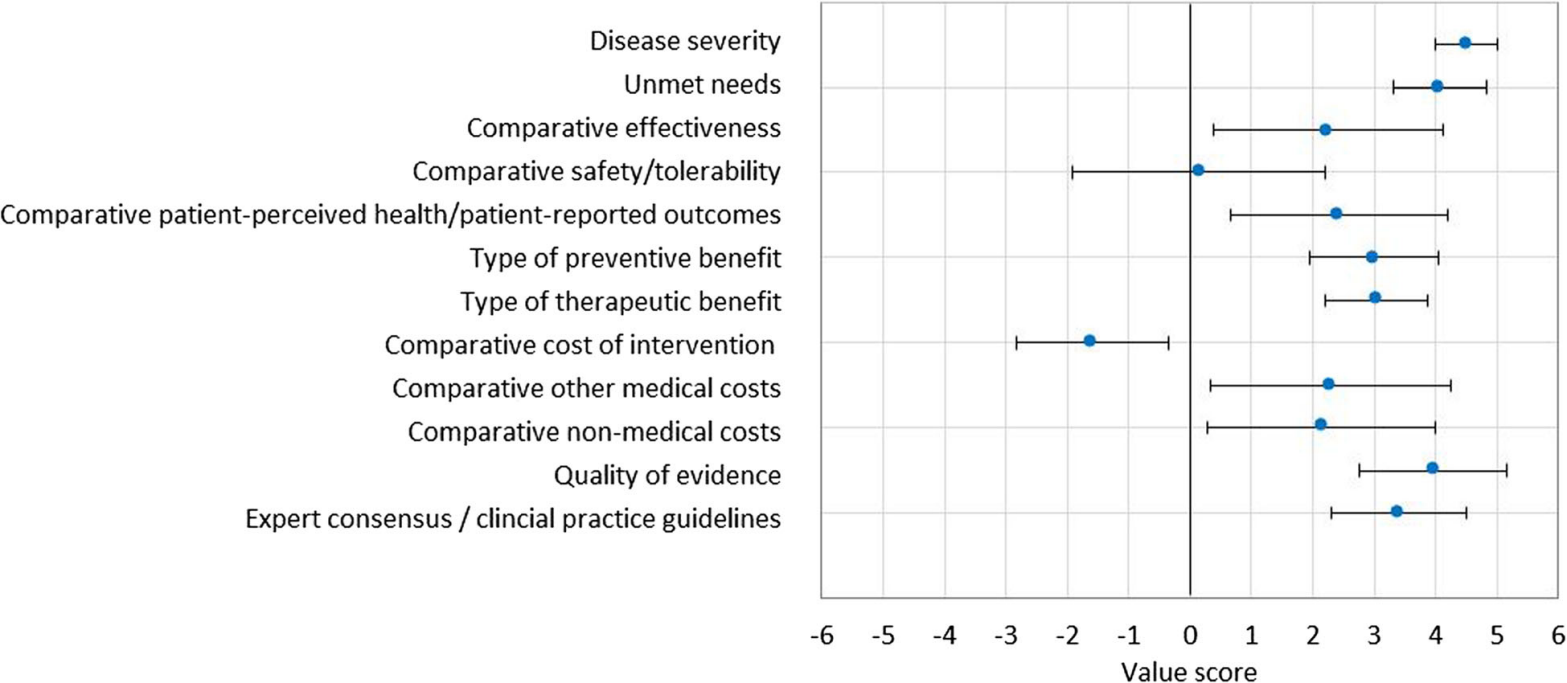
Scoring of value contribution of selexipag to PAH treatment compared to iloprost according to the adapted MCDA framework quantitative criteria. Mean (SD) scores assigned to each quantitative criterion by experts are shown. Error bars show standard deviations across the 28 study participants. A constructed, cardinal scoring scale was used, ranging from 0 to 5 for non-comparative and from − 5 to 5 for comparative criteria, respectively

The scores resulting from both phases of the study for each criterion were analysed separately. There were no significant differences between the score means from both studies for any of the analysed criteria (95% CI *p* > 0.05). Thus, both samples were combined into a single database and further analysed as shown in Fig. 2. Only statistically significant differences reported across participants’ professional profiles were mentioned.

“Severity of disease” scored 4.5 ± 0.5 (mean score ± SD) on a scale of 0 to 5, reflecting experts’ perception of its impact on mortality and patient’s quality of life. Although all participants assigned a high score value to this criterion, statistically significant differences were only reported between scores from clinicians and healthcare decision makers (mean score 4.81 vs 4.17 respectively; 95% CI: 0.04 to 1.26; *p* = 0.03). The “Unmet needs” criterion scored 4.1 ± 0.8 on a scale of 0 to 5, as all participants agreed that PAH is a condition with important unmet needs, mainly with regards to the lack of curative and more effective treatments, the need for earlier diagnosis and for treatments with a more convenient route of administration and posology.

Selexipag’s “comparative effectiveness” versus iloprost scored 2.3 ± 1.9 on a scale of − 5 to 5. Most participants assigned a positive score to this criterion (19 out of 21). Experts pointed out that the positive score in the “comparative efficacy” criterion for selexipag was due to the robust data obtained in clinical trials compared to placebo (no direct comparative data for selexipag versus iloprost were available). There were statistically significant differences in opinions for this criterion between clinicians and hospital pharmacists (3.3 vs 1, respectively, 95% CI: 0.40 to 4.15; *p* = 0.01). No differences in terms of “comparative safety/tolerability” were reported, as this criterion scored 0.1 ± 2.1 on a scale of − 5 to 5, due to the similar rate of adverse events reported with selexipag and inhaled iloprost. “Comparative patient reported outcomes (PROs)” scored 2.4 ± 1.8 on a scale of − 5 to 5. Selexipag was perceived as a drug capable of providing value in terms of patient’s quality of life compared to inhaled iloprost due to the convenience of its route of administration and posology: orally for selexipag versus inhaled for iloprost, and the dosing schedule (two times per day vs 6–9 times per day, respectively [25, 26]). Some experts pointed out that the use of two different questionnaires in clinical trials (the CAMPHOR questionnaire [27] for selexipag and the EQ-5D VAS [28] for iloprost) did not allow the comparison of quality of life data for both drugs.

Study participants scored the criterion “preventive benefit” positively, as it scored 3.0 ± 1.1 on a scale of 0 to 5, due to its mechanism of action, with the potential to stabilise the disease, reduce morbidity-mortality events and decrease the need to progress to more inconvenient treatments. The “therapeutic benefit” of selexipag was also scored positively across all participants (3.0 ± 0.8 on a scale of 0 to 5), based on the impact on the delay of the progression of the disease, the therapeutic convenience due to its oral administration and the impact on reducing the number of hospitalisations.. The “comparative cost” of selexipag was analysed only considering the scores from the second phase of the study, when an official approved price for selexipag in Spain was available. “Comparative cost” scored − 1.6 ± 1.6 on a scale of − 5 to 5. This negative score for selexipag was due to the higher price of selexipag vs inhaled iloprost (incremental cost of selexipag 9.8%, ex-factory price per day 128.8 € vs 117.3 €, respectively, according to the mean dosage recommended in the summary of product characteristics of both drugs (2 doses/day for selexipag and 7.5 inhalations/day for iloprost) [25, 26]). Three scoring results from the second phase in which participants scored selexipag’s higher price positively were treated as inconsistences. Experts scored selexipag positively for the “comparative other medical costs” (2.3 ± 2.0 on a scale of − 5 to 5), reflecting their perception of the economic benefits obtained due to savings on healthcare resources resulting from treatment with selexipag compared to use of iloprost. However, hospital pharmacists noted these savings had not been quantified and are not usually considered at time of inclusion of a drug into a hospital formulary. Selexipag is perceived as a therapeutic option that can produce savings in “non-medical costs” compared to iloprost (this criterion scored 2.1 ± 1.9 on a scale of − 5 to 5), because it can improve the daily life of patients with PAH (e.g. better quality of life, reduced cost of carers). Thus, most participants considered that the cost difference between the two products would be acceptable for payers, due to the low prevalence of the disease, the potential cost-offsets derived from the reduction of other medical costs, the perceived improvement of patient’s quality of life and other expected clinical benefits resulting from treatment with selexipag.

The quality of the evidence supporting selexipag was perceived as very good by study participants (this criterion scored 4.0 ± 1.2; on a scale of 0 to 5). Participants recognised the quality of both the design and the analysis of the clinical variables from the GRIPHON pivotal trial. There were statistically significant differences in the scores between clinicians and hospital pharmacists (4.8 vs 3.6 respectively; 95% CI: 0.01 to 2.4; *p* = 0.04) and between clinicians and healthcare decision makers (4.8 vs 3 respectively; 95% CI: 0.01 to 0.4; *p* = 0.007). Some experts suggested the combined morbidity-mortality variable should be presented separately in study results, because the main unmet clinical need in PAH remains improvement in survival. Also, the comparison to placebo was considered as a limitation by some participants. The inclusion of selexipag in clinical practice guidelines as a reference for PAH treatment [1] was considered positively (this criterion scored 3.4 ± 1.1, on a scale of 0 to 5) (Table 2).

**Table 2:** Mean scores, standard deviations and main comments from study participants for each criteria of the MCDA framework

MCDA framework criteria	Mean score ± Standard Deviation	Main comments from participants
Disease severity	4.5 ± 0.5 on a scale of 0 to 5	• “The impact of PAH on patient’s health (in the most severe cases) and quality of life and family environment (even in less advanced cases or responders to treatment) is severe, given the irreversible nature of the process, often known by patients / family, which leads to a pessimistic view about it.” • “Interference, often serious, when performing normal daily activities”
Unmet needs	4.1 ± 0.8 on a scale of 0 to 5	• “More effective and selective drugs are needed that reduce mortality and disease progression in clinical trials and in real life. Greater comfort than prostacyclines, so an oral treatment is welcome” • “Currently available drugs that act on the prostacyclin route are uncomfortable, and they require time and training from patients and caregivers and interfere with the proceeding of a normal daily activity.”
Comparative efficacy/effectiveness	2.3 ± 1.9 on a scale of −5 to 5	• “Although there is no data available for all the variables in the studies conducted with iloprost that allow comparison with selexipag, it seems that better results are seen corresponding to “death by any event.” First Event. However, I believe that the impossibility of comparing more extensively with other alternatives limits the interpretation of results.” • “The results presented favour the GRIPHON study of selexipag. Not only for the inclusion of a greater number of patients (up to five times more) but also for the design and choice of the primary endpoints. The designation of morbi-mortality parameters (progression, death, hospitalization, need for IV therapy, ...) make the trial more appropriate to the actual practice, with greater statistical power and conforms to the guidelines designated in the PAH symposium at Dana Point 2007.”
Comparative safety/tolerability	0.1 ± 2.1 on a scale of − 5 to 5	• “I think that the lower proportion of patients who died due to “any cause”, who had to interrupt the medication and who had potentially more severe undesirable effects (syncope) in the selexipag group, could be an element to be considered in the choice of selexipag with respect to iloprost.” • “It seems that iloprost has fewer adverse effects than what is mentioned, although similar at a general level.”
Comparative patient-perceived health/patient reported outcomes (PRO)	2.4 ± 1.8 on a scale of − 5 to 5	• “Since there is no data regarding the groups treated with iloprost, even assuming that these are not studies that by their design allow direct comparison, it is not possible for me to clearly opt for any, except for the aspects related to the administration of the drugs (posology, manipulation of the inhalation device...), which evidently can suppose an interference in the quality of life of the patient derived from the greater complexity in the case of iloprost.” • “The limitation imposed by the inhalation of iloprost up to 9 times a day is important, uncomfortable and makes the patient self-conscious” • “An oral dosage twice a day clearly improves the quality of life and the autonomy of the patient.”
Type of preventive benefit	3.0 ± 1.1 on a scale of 0 to 5	• “The reduction of mortality due to any event, and the need for hospitalization, especially taking into account the ease of administration with respect to prostanoids, confers a high therapeutic value in my opinion.” • “It does not prevent the disease, although, it stabilises and delays the appearance of new events of morbidity and mortality in relation to the disease.”
Type of therapeutic benefit	3.0 ± 0.8 on a scale of 0 to 5	• “The improvement in the time of progression of the disease and the increased convenience of administration are, in my opinion, favourable criteria to selexipag, especially in relation to prostanoids (aerosolized or parenteral), although the lack of effect on mortality continues to limit its therapeutic efficacy.” • “It does not cure, it stabilizes and probably helps to chronify the disease.”
Comparative cost of intervention	−1.6 ± 1.6 on a scale of − 5 to 5	• “The annual cost is substantially higher in the case of selexipag, which undoubtedly hinders its acceptance by the paying entities, although the convenience in its administration, with favorable repercussion on the quality of life of the patient and caregivers, as well as improvement in the evolutionary course of the disease (with reduced costs related to, for example, the need for hospitalization) should be arguments to be considered in the negotiation with the health authorities” • “The economic cost of selexipag is 10% more than that of inhaled iloprost, which does not seem excessive, considering the ease of application.”
Comparative other medical costs	2.3 ± 2.0 on a scale of − 5 to 5	• “The main benefit of selexipag in terms of costs lies in its potential effect on the reduction of indirect costs, such as the need for hospitalizations, emergency visits, or other specific techniques or treatments. Although these costs are difficult to quantify for hospital pharmacies, they are one of the main problems in chronic diseases.” • “The reason for the assigned score is purely estimative, since there are no cost-efficiency studies, neither with selexipag nor with any other therapeutic alternative.” • “The economic impact of a drug is measured not only by its initial cost or investment, but by its ability to save in other aspects, so, a priori, selexipag seems to have a better impact on costs.”
Comparative other non-medical costs	2.1 ± 1.9 on a scale of − 5 to 5	• “The ease of administration / therapeutic compliance with selexipag could represent significant economic savings in the occupational and social sphere of patients.” • “Difficult to estimate the impact on productivity, since the majority of patient candidates for selexipag or iloprost have a recognized legal incapacity status.”
Quality of evidence	4.0 ± 1.2; on a scale of 0 to 5	• “I believe that it strictly meets the quality criteria required for a clinical trial, especially given the difficulties of including patients as it is a low prevalence disease.” • “I believe that the Griphon study is one of the best designed, with the largest number of patients included, with a broad spectrum of basic treatments (similar to the usual practice) and with one of the longest follow-up periods to date” • “The scientific evidence provided by the Griphon study is now unquestionable, well above the existing data for many other medications and marks a new paradigm within clinical trials in pulmonary hypertension.”
Expert consensus/clinical practice guidelines (CPG)	3.4 ± 1.1, on a scale of 0 to 5	• “The current guideline recommendations place selexipag in both functional class II and III of the WHO, with a high level of evidence (IA). However, no distinction is made with respect to other oral drugs (endothelin antagonists or phosphodiesterase inhibitors).” • “The real distinction is made with respect to analogous drugs (prostacyclines), with an introduction in earlier stages (functional class II), as well as a higher level of evidence.” • “In the clinical guidelines of clinical practice, selexipag is recommended with the same degree of indication (degree I) as the rest of oral treatments with grade B evidence, similar to riociguat or macitentan.”

**Fig. 3:**
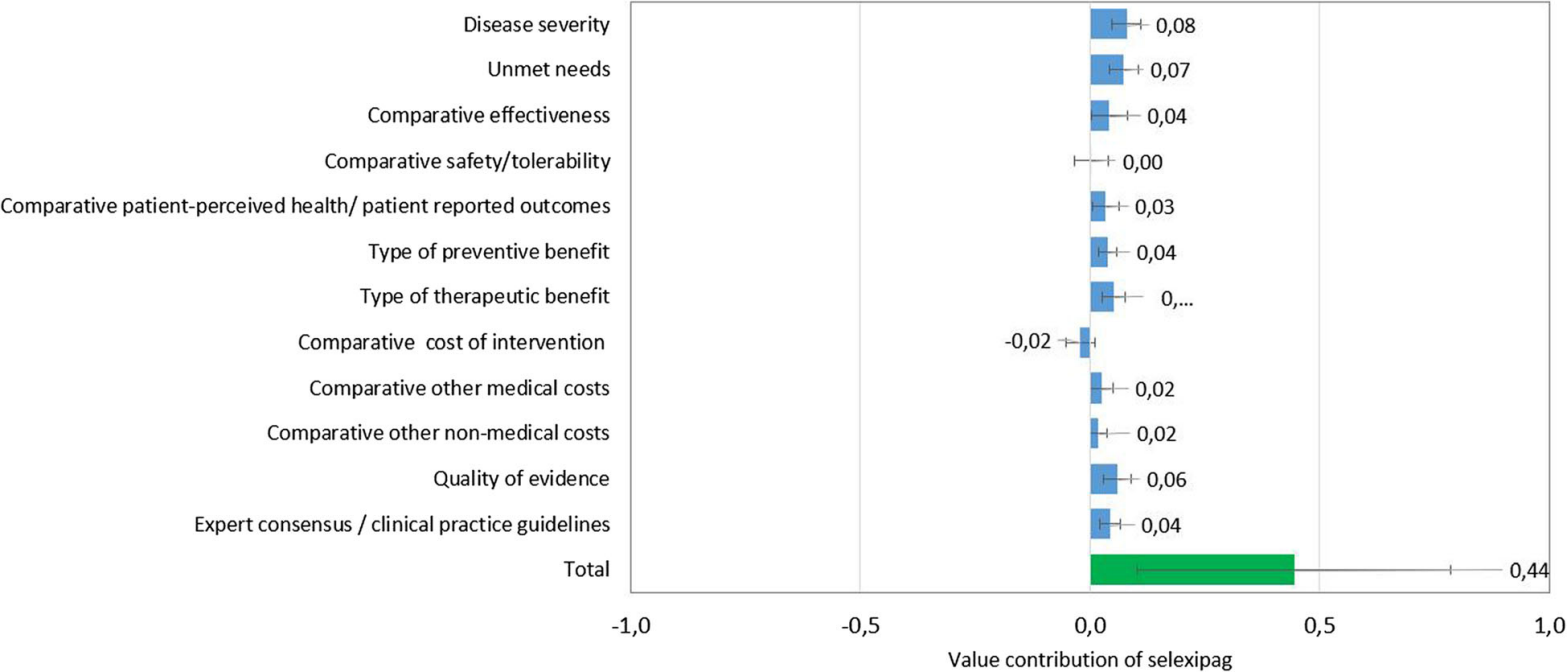
Weighted value contribution of selexipag for the treatment of PAH compared to iloprost according to quantitative criteria of the adapted MCDA framework. Mean value contributions of each quantitative criterion and overall MCDA value estimates for selexipag in the PAH treatment are shown. Error bars show standard deviations across the 28 study participants

Figure 3 shows the overall value contribution of selexipag to each criterion for PAH treatment when adjusting the values of the scores obtained during the evaluation to the relative importance assigned to each criterion (weighting).

**Fig. 4:**
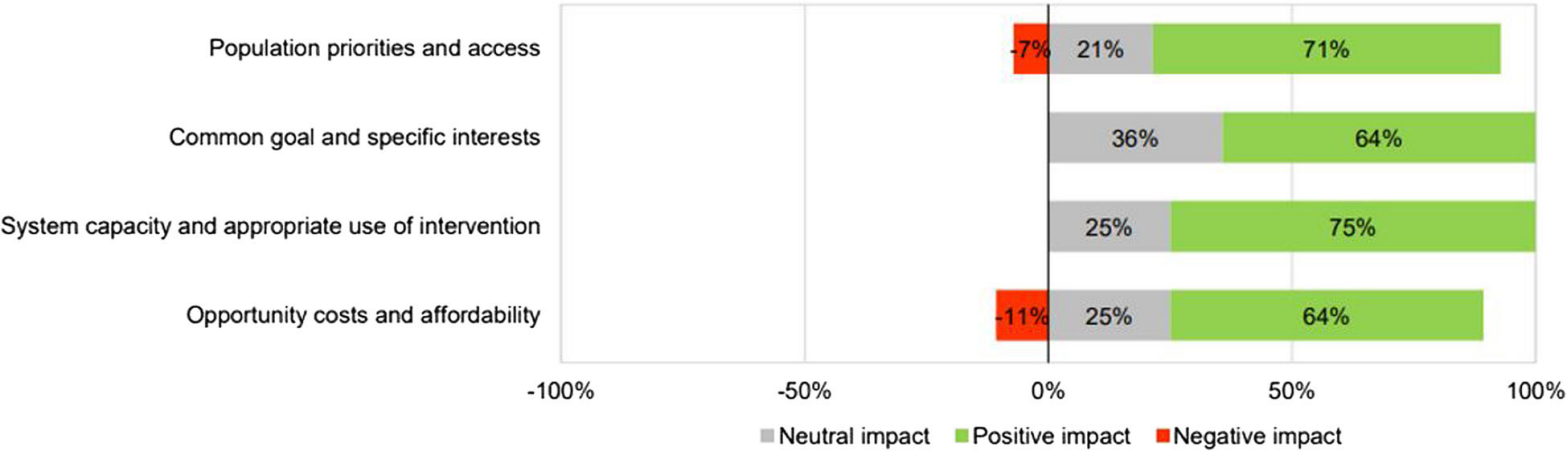
Percentage of participants who considered that the incorporation of selexipag for the treatment of PAH in Spain would have some type of impact with respect to the contextual criteria of the adapted MCDA framework

Assessment of the qualitative criteria from the adapted MCDA framework was positive for all criteria (Fig. 4). Participants scored the qualitative criteria in terms of positive, neutral or negative impact of selexipag for each criterion. Selexipag was perceived as a valuable therapeutic alternative that would have a positive impact in terms of “priority access for population”, according to 71% of study participants (7% perceived it would have a negative impact and 22% a neutral impact), as it would be expected that the classification of PAH as a rare disease and the severity of the disease would favour the incorporation of innovative treatments. However, even though rare disease treatment is a priority for the Spanish national healthcare system, access to new treatments in practice can be slow, according to the opinion of some participants. A total of 64% of experts agreed that selexipag was aligned with the “common goals and specific interests” item of the MCDA framework and that selexipag’s access could be supported by scientific societies, patient associations and other professional groups because it matches the unmet needs identified for the disease. Although participants noted there is multidisciplinary interest from healthcare professionals in the approval of new drugs for diseases with high unmet needs, 36% participants considered there was no impact on this criterion considering the limited prevalence of PAH, which would dilute the specific pressures for inclusion of selexipag into drug formularies. Unlike for other drugs administered by intravenous injection or inhaled, study participants considered treatment with selexipag will not require specific training for patients and healthcare professionals involved in PAH. Thus, the impact on “system capacity and appropriate use of intervention” was considered positively by 75% of participants and with neutral impact for the remaining 25%.

The experts perceived selexipag’s budget impact as negative, due to the higher acquisition cost of selexipag compared to iloprost and the potential positive impact on disease prognosis arising from treatment with selexipag, which will, in turn, increase its prevalence. Although the budget impact was perceived negatively, a total of 64% of participants agreed that the use of selexipag would have a positive impact on the “opportunity costs and affordability” criterion, because of treatment derived clinical benefits and savings in other medical resources (i.e.: less hospital visits, less hospitalisations due to increased efficacy, training from healthcare professionals to patients on the treatment administration). Twenty-five per cent (25%) of participants considered selexipag to have a neutral impact on this criterion and 11% considered selexipag to have a negative impact due to the perception that selexipag would increase the hospital pharmacy budget. In summary, experts believe that treatment of PAH with selexipag would be aligned with the Spanish national healthcare system priorities and the system would be prepared for management of selexipag in real clinical practice.

## Discussion

The value contribution of selexipag to PAH treatment and compared to inhaled iloprost was assessed through reflective MCDA by a multidisciplinary panel of people involved in the management of PAH treatments and decision-making in Spain who scored the product’s evidence matrix, which allowed a holistic value determination of selexipag, including the specific context of its appraisal in Spain.

The development and use of an on-line study platform allowed the assessment by a large enough number of regionally wide-spread experts, adding to the robustness of the study. We have been able to collect quantitative and qualitative data, including group discussions in the face-to-face panel and a broad number of individual comments in both phases of the study, reflecting the reasoning behind each participant’s scoring. To our knowledge, this represents the first example of the use of an on-line platform for a study using reflective MCDA methodology for healthcare evaluation in Spain. We have only identified one study which uses an on-line MCDA platform, conducted by Garau et al. in Italy [29].

The criteria that contributed the most positively to the overall value of selexipag were “disease severity”, “unmet needs” and “quality of evidence”. The only criteria that contributed negatively to the product’s value was the “comparative cost of intervention”. This last result was not surprising as selexipag is a more expensive drug than inhaled iloprost, although, according to the experts, may have some potential savings in medical and non-medical costs.

When considering contextual criteria, selexipag was considered a treatment for PAH aligned with the Spanish national healthcare system’s priorities. Study participants considered that no major barriers to patient access should be expected, as PAH is considered a very severe disease with high unmet needs in the context specific to prostacyclin pathway treatments. It was also noted that there is a multidisciplinary interest across healthcare professionals for the approval of new effective treatments for PAH. Experts agreed that the Spanish healthcare system would be prepared for the management of selexipag in real practice and it would have a positive impact on patients.

Evaluation of the value innovative products in Spain is carried out sequentially at different levels with specific objectives in each one of them: national level (focused on pricing & reimbursement decisions and clinical positioning of the drug), regional level (focused on resources management) and local level (focused on hospital access, prioritisation and utilisation criteria) [30].

In general terms, value assessment of medical treatments in Spain is still based mainly on efficacy, safety and cost criteria. While healthcare evaluation bodies acknowledge the importance of considering additional criteria in their decision-making processes, this is currently neither formally established nor standardised, resulting in the practical application of different approaches at different levels [30].

Official pricing and reimbursement criteria used to include a new drug into the Spanish public reimbursement system, are defined in the article 92.1 of the Royal Decree Law 1/2015 [31]. These criteria can be easily related to those included in MCDA frameworks, showing the importance of a complete value assessment of the product, in order to reduce evaluator and decision makers’ uncertainties: severity of the disease (relates to severity of disease criterion), specific needs of certain groups (unmet needs and contextual criteria), therapeutic and social value and incremental clinical benefit of the medication according to its cost-effectiveness ratio (clinical comparative criteria, therapeutic and preventive benefit, clinical evidence, inclusion in clinical guidelines and contextual criteria), rationalisation of public spending and budgetary impact on the national healthcare system (comparative economic criteria and contextual criteria), existence of alternatives for the same conditions at a lower price or lower treatment cost (unmet needs and comparative cost of intervention criteria) and the drug’s degree of innovation (therapeutic benefit criterion). Spanish evaluators and decision makers have already considered the use of MCDA frameworks (and their value criteria) as a complete and useful tool, feasible to be used for drug evaluation and decision-making in Spain [32].

This study provides a multistakeholder value assessment of selexipag, including criteria that can help evaluators and decision makers to assess the overall added value provided by the product in the PAH setting in Spain. MCDA methodology can provide a standardised and holistic value assessment that is valid at all levels, which goes beyond efficacy, safety and cost, especially relevant in complex disease areas (e.g. rare diseases).

This study has some limitations. For the evaluation of some comparative criteria, the available evidence for selexipag and iloprost at the time of the study was limited. Indirect comparisons had to be performed since evidence for both drugs came from studies with very different populations, study design, analytical methodologies and outcome variables. In addition, iloprost has been marketed in Spain since 2004 while selexipag, since May 2017. Thus, healthcare professionals specialised in PAH can have a wide practical experience with iloprost and the comparison between the two drugs could be influenced by such previous experience.

The novel design of this study solved one of the major limitations that MCDA value appraisal studies usually present: the small number of experts involved in the study [33]. The sufficiently large number of participants allowed the appraisal to be representative for the whole country and was able to consider a large variety of stakeholder’s perspectives and reflections, contributing to study results validity and relevance.

## Conclusions

Selexipag is a selective IP receptor agonist which was the first oral drug approved in Europe for the long-term treatment of PAH in adult patients with WHO functional class (FC) II–III. The participants of the study perceived selexipag as an intervention with a positive value contribution to PAH treatment, that is indicated for a severe rare disease with perceived high unmet needs (mainly with regards to the lack of curative and more effective treatments and a more convenient route of administration and posology), supported by high quality clinical evidence and that has demonstrated improvements in efficacy and patient reported outcomes, with limited additional added value in terms of safety, and some additional costs when compared to inhaled iloprost.

The holistic value contribution of selexipag was successfully assessed by a large multidisciplinary panel of relevant stakeholders for drug evaluation and healthcare decision making in Spain using a web-based extended MCDA form.

To our knowledge, this represents the first example of the use of an on-line platform for a study using reflective MCDA methodology in Spain.

## Abbreviations

AEMPS: Agencia Española de Medicamentos y Productos Sanitarios
AEs: Adverse effects
CI: Confidence interval
EMA: European Medicines Agency
EPAR: European Public Assessment Report
EQ-5D: quality of life questionnaire- 5 dimensions
ERA: Endothelin receptor agonist
EVIDEM: Evidence and values impact on decision making
FC: Functional class
IP: Prostacyclin receptor
MCDA: Multi-criteria decision analysis
NO: Nitric oxide
PAH: Pulmonary Arterial Hypertension
PDE: Phosphodiesterase
SD: Standard deviation
VAS: Visual analogic scale
VS: Versus
